# Patients’ and caregivers’ perception of multidimensional and palliative care in amyotrophic lateral sclerosis – protocol of a German multicentre study

**DOI:** 10.1186/s42466-024-00328-1

**Published:** 2024-07-04

**Authors:** Katharina Linse, Constanze Weber, Peter Reilich, Florian Schöberl, Matthias Boentert, Susanne Petri, Annekathrin Rödiger, Andreas Posa, Markus Otto, Joachim Wolf, Daniel Zeller, Robert Brunkhorst, Jan Koch, Andreas Hermann, Julian Großkreutz, Carsten Schröter, Martin Groß, Paul Lingor, Gerrit Machetanz, Luisa Semmler, Johannes Dorst, Dorothée Lulé, Albert Ludolph, Thomas Meyer, André Maier, Moritz Metelmann, Martin Regensburger, Jürgen Winkler, Berthold Schrank, Zacharias Kohl, Tim Hagenacker, Svenja Brakemeier, Ute Weyen, Markus Weiler, Stefan Lorenzl, Sarah Bublitz, Patrick Weydt, Torsten Grehl, Sylvia Kotterba, Hanna-Sophie Lapp, Maren Freigang, Maximilian Vidovic, Elisa Aust, René Günther

**Affiliations:** 1 University Hospital Carl Gustav Carus at Technische Universität Dresden, Fetscherstrasse 74, 01307 Dresden, Germany; 2 German Center for Neurodegenerative Diseases Dresden, Tatzberg 41, 01307 Dresden, Germany; 3 Department of Neurology with Friedrich-Baur-Institute, University Hospital, Ludwig-Maximilians-University (LMU), Marchioninistrasse 15, 81377 Munich, München Germany; 4 University Hospital Münster (UKM), Albert-Schweitzer-Campus 1, 48149 Münster, Germany; 5 Department of Medicine, UKM-Marienhospital Steinfurt, Mauritiusstr. 5, 48565 Steinfurt, Germany; 6 Hannover Medical School, Carl-Neuberg-Strasse 1, 30625 Hannover, Germany; 7 University Hospital Jena, Am Klinikum 1, 07747 Jena, Germany; 8 University Hospital Halle, Ernst-Grube-Strasse 40, 06120 Halle (Saale), Germany; 9 Deaconess Hospital Mannheim, Speyerer Strasse 91-93, 68163 Mannheim, Germany; 10 University Hospital Würzburg, Josef-Schneider-Strasse 11, 97080 Würzburg, Germany; 11 University Hospital RWTH Aachen, Pauwelsstrasse 30, 52074 Aachen, Germany; 12 University Medical Center Göttingen, Robert-Koch-Strasse 40, 37075 Göttingen, Germany; 13 University Medical Center Rostock, Translational Neurodegeneration Section „Albrecht Kossel“, Gehlsheimer Strasse 20, 18147 Rostock, Germany; 14 German Center for Neurodegenerative Diseases Rostock/Greifswald, Gehlsheimer Strasse 20, 18147 Rostock, Germany; 15 University of Lübeck, Ratzeburger Allee 160, 23538 Lübeck, Germany; 16 Hospital Hoher Meißner, Hardtstrasse 36, 37242 Bad Sooden-Allendorf, Germany; 17 Evangelic Hospital Oldenburg, Steinweg 13-17, 26122 Oldenburg, Germany; 18 Klinikum rechts der Isar, TUM School of Medicine and Health, Technical University of Munich, Ismaninger Strasse 22, 81675 Munich, Germany; 19 German Center for Neurodegenerative Diseases Munich, Feodor-Lynen-Strasse 17, 81377 Munich, Germany; 20 University Hospital Ulm, Oberer Eselsberg 45, 89081 Ulm, Germany; 21 Charité – Universitätsmedizin Berlin, Augustenburger Platz 1, 13353 Berlin, Germany; 22 University Hospital Leipzig, Liebigstrasse 20, 04103 Leipzig, Germany; 23 University Hospital Erlangen, Schwabachanlage 6, 91054 Erlangen, Germany; 24 DKD Helios Clinic Wiesbaden, Aukammallee 33, 65191 Wiesbaden, Germany; 25 University Hospital Regensburg, Universitätsstrasse 84, 93053 Regensburg, Germany; 26 Department of Neurology, Center for Translational Neuro- and Behavioral Sciences (C-TNBS), University Hospital Essen, Hufelandstr. 55, 45147 Essen, Germany; 27 BG University Hospital Bochum, Bürkle de la Camp-Platz 1, 44789 Bochum, Germany; 28 Department of Neurology, Heidelberg University Hospital, Im Neuenheimer Feld 400, 69120 Heidelberg, Germany; 29 Hospital Agatharied, Norbert-Kerkel-Platz, 83734 Hausham, Germany; 30 Institute of Palliative Care and Institute of Nursing Science, Paracelsus Medical University, Salzburg, Austria; 31 Department of Palliative Medicine, University Hospital, Ludwig-Maximilians-University (LMU), Marchioninistrasse 15, 81377 Munich, München Germany; 32 University Hospital Bonn, Venusberg Campus 1, 53127 Bonn, Germany; 33 Alfried-Krupp-Hospital Essen, Alfried-Krupp-Strasse 21, 45131 Essen, Germany; 34 Hospital Leer, Augustenstrasse 35-37, 26789 Leer, Germany

**Keywords:** Amyotrophic lateral sclerosis, Caregiver burden, Palliative care, Quality of care, Motor neuron disease, Psychosocial care

## Abstract

**Introduction:**

Amyotrophic lateral sclerosis (ALS) is an inevitably fatal condition that leads to a progressive loss of physical functioning, which results in a high psychosocial burden and organizational challenges related to medical care. Multidimensional and multiprofessional care is advised to meet the complex needs of patients and their families. Many healthcare systems, including Germany, may not be able to meet these needs because non-medical services such as psychological support or social counselling are not regularly included in the care of patients with ALS (pwALS). Specialised neuropalliative care is not routinely implemented nor widely available. Caregivers of pwALS are also highly burdened, but there is still a lack of support services for them.

**Methods:**

This project aims to assess the perceptions and satisfaction with ALS care in Germany in pwALS and their caregivers. This will be achieved by means of a cross-sectional, multicentre survey. The examination will assess, to which extend the patients’ needs in the six domains of physical, psychological, social, spiritual, practical and informational are being met by current care structures. This assessment will be linked to mental well-being, subjective quality of life, attitudes toward life-sustaining measures and physician-assisted suicide, and caregiver burden. The study aims to recruit 500 participants from nationwide ALS centres in order to draw comprehensive conclusions for Germany. A total of 29 centres, mostly acquired via the clinical and scientific German Network for Motor Neuron Diseases (MND-NET), will take part in the project, 25 of which have already started recruitment.

**Perspective:**

It is intended to provide data-based starting points on how current practice of care in Germany is perceived pwALS and their caregivers and how it can be improved according to their needs. Planning and initiation of the study has been completed.

**Trial registration:**

The study is registered at ClinicalTrails.gov; NCT06418646

## Introduction

Amyotrophic lateral sclerosis (ALS) is the most prevalent adult onset motor neuron disease. Currently, over 5,000 people affected by ALS live in Germany [1]. Due to the progressive degeneration of motor neurons, ALS leads to a continuous decline in voluntary muscle functioning, resulting in the loss of mobility as well as in disabilities of speech and swallowing. The impairment of respiratory function due to the involvement of the respiratory muscles usually leads to death within 2-4 years after symptom onset, unless patients opt for invasive ventilation [2].

**Fig. 1:**
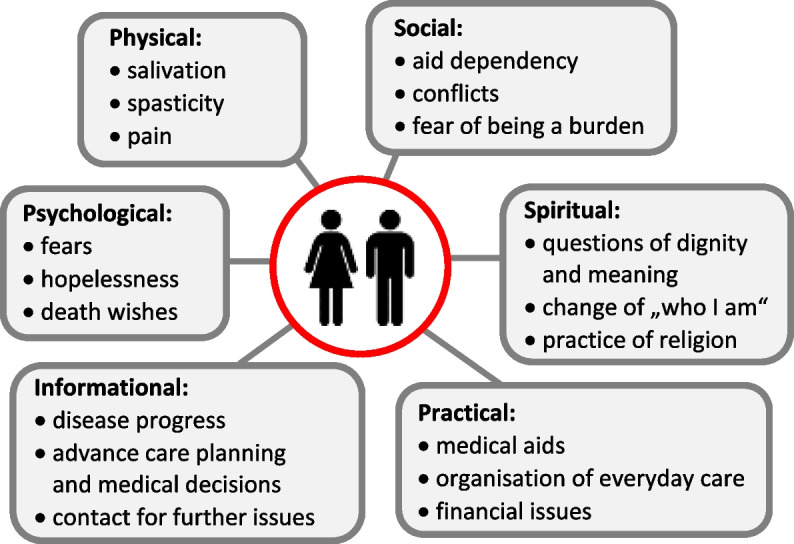
Examples of the needs and suffering of pwALS in six domains, based on the Supportive Care Framework according to Fitch [13]. This model served as basis for the newly developed patient questionnaire

ALS care is characterised by two central factors, which are closely intertwined:

First, patients’ disease burden, their needs and the corresponding requirements for professional care are dynamic and involve different domains, see Fig. 1 [3]. Psychosocial and socio-medical aspects have significant impact on patients’ QoL: Naturally, health related QoL worsens throughout the disease course, however, patients’ subjective QoL is more strongly associated with psychological factors such as anxiety and depression [4]. In addition, QoL is related to the provision of appropriate medical aids that support patient autonomy and compensate for deficits [5]. Although data is limited, multidimensional, whole-person support has been shown to enhance some aspects of patients’ QoL [6]. Individualised advance care planning is another important issue in ALS care, including decisions about gastrostomy, non-invasive as well as tracheostomy invasive ventilation and cardiopulmonary resuscitation. Advance care planning is recommended to commence timely and be revisited periodically, given that patient preferences may significantly change over time [7]. This process is time consuming and demands a high level of expertise in dealing with the end-stage of ALS, along with sensitivity towards the patient’s background, personality and individual values and beliefs.

Moreover, it is well-known that family caregivers of patients with ALS (pwALS) experience burden at multiple levels, are highly stressed and sometimes report a lower QoL than the patients themselves [8]. Relief for family caregivers can be provided, for example by access to psychosocial counselling and support, 24/7 availability of support in the event of a sudden crisis, or by setting up respite care if necessary [6].

Given these complex and dynamic requirements, highly specialised, *multidimensional and multiprofessional care* is required, as outlined in the current ALS treatment guidelines [9]. International studies suggest that this approach offers advantages in terms of survival time, decision-making, QoL and patient satisfaction with care [10–12].

Second, as there is still no curative treatment available for ALS, the therapeutic approach is essentially palliative from the time of diagnosis. The primary treatment goal is to alleviate symptom burden and enhance quality of life (QoL) throughout the course of the disease, by providing *Palliative Care*. Palliative Care is multidimensional by definition and involves the alleviation of as well physical as also psychosocial or spiritual suffering in the context of a life-limiting disease, encompassing the family system and focusing on the individual QoL and needs of both patients and caregivers (Table 1). Basically, the scientific literature advocates the early integration of *specialised* palliative care (SPC) for individuals who experience a high symptom burden - if necessary from the time of diagnosis [6]. While systematic studies are scarce, SPC is believed to be linked to improved symptom management during the course of the disease and at the end of life. Furthermore, it is associated with better QoL and enhanced communication both with and within the affected families, resulting in positive effects on patient-centred treatment planning in ALS [6, 14–16]. For oncological diseases, SPC has also been shown to reduce psychosocial distress such as anxiety and the feeling of being a burden [17]. Meanwhile, the optimal integration of SPC in ALS care remains unclear and standardised pathways for practice have not yet been established. In accordance, the above-cited literature on SPC in ALS describes different arrangements, such as integrating an SPC expert into an ALS-specialised team or systematically referring pwALS to SPC providers, guided by predefined trigger-points. In Germany, specialised outpatient palliative care is the most common way of integrating SPC in ALS care. However, in practice, this is often limited to the last weeks of life, contradicting the ALS treatment guidelines [18–20]. The requirements for the involvement of SPC providers, its maximum duration as well as its effectiveness on patient-centred outcomes vary between the federal states of Germany [21].Timely availability, neurological knowledge and awareness of the special needs of pwALS also vary between providers.

**Table 1:** Definitions of three different specifications of Palliative Care (as mentioned in the text) und potential providers in the context of ALS care in Germany

**Concept**	**Situation**	**Provider**
**Primary Palliative Care**	No particular complexity of the situation	• General practitioners and Specialist physicians (some services can only be invoiced if special qualifications can be evidenced)
**Specialised Palliative Care**	Complex situation and/or high symptom burden	• Specialised outpatient palliative care • Palliative care units and day clinics
**NeuroPalliative Care (as a part of SPC**, see [20]**)**	Complex situation and/or high symptom burden; predominantly neurological symptoms and treatment requires neurological expertise	• Neuropalliative care units • Palliative care units with access to neurological expertise or vice versa • Specialised ALS centres

*SPC* Specialised palliative care

SPC has so far mainly focused on oncological diseases. Therefore, the new German treatment guidelines for *neuro*palliative patients specify recommendations for SPC in neurological disorders, termed *neuro*palliative care (NPC; see table 1), taking into account the specific needs of patients with neurological diseases. However, apart from a few flagship projects (https://neurologie.charite.de/leistungen/klinische_schwerpunkte/neuropalliativecare/; https://www.khagatharied.de/die-medizin-2/neurologie/als-home-care/), NPC structures are not yet established in Germany. Specialised ALS centres are mentioned as providers of NPC in the guidelines, provided that they have experience in caring for patients in their last phase of life [20].

In practice, ALS centres devote a great amount of time to NPC, including repetitive counselling with regard to end-of-life decisions, advance care planning, and provision of appropriate pharmacological symptom treatment and medical aids, complemented by unscheduled telephone- or video-calls in case of crisis and the provision of emotional support. However:

1. Special qualifications in palliative care are not mandatory and the level of individual experience varies
2. Structural and personnel requirements for treating patients with a high and possibly dynamically exacerbating palliative symptom burden are not given, such as 24/7 availability of the team or the possibility of home visits when needed
3. Most importantly, adequate cost-effective compensation for these services is not provided, particularly since non-medical professions are completely excluded from the reimbursement process – in crucial contrast to Specialised outpatient palliative care.

In summary, there are large discrepancies between the current standard care structures in Germany and the well-known imperative for multidimensional, palliative care of pwALS and their caregivers. Neither the multidimensionality of patients’ needs is displayed in standard care, as non-medical services such as psychological support or social counselling are not regularly included or can be reimbursed; nor is the need for medically specialised NPC. Additionally, there is a lack of support services for caregivers.

International studies suggest that subjective deficiencies in care can significantly impact the well-being of patients: they mediate the relationship between disease severity and QoL [22] and have an impact on decision-making regarding life-prolonging measures [23]. In an interview study, a large proportion of caregivers reported deficits in the professional care provided, which was associated with stronger caregiver burden and health impairments–some of which persist for years after the patients’ death [18, 24]. Furthermore, psychological well-being of caregivers is an important factor influencing if patients can live and be cared for at their home [6, 8]. Finally, yet importantly, a legal regulation of physician-assisted suicide is currently being discussed in Germany–an option that necessitates optimised and accessible palliative care services at the same time [25]. This is crucial to avoid suicides committed as a consequence of physical or psychological suffering that could be at least partially alleviated.

## Methods

### Aim of the study

The primary aim of the project is to investigate how pwALS and their caregivers perceive and are satisfied with different aspects of ALS care in Germany, focusing on their potential needs in the six domains displayed in Fig. 1. So far, it has not been recorded systematically to what extent the structural weaknesses of ALS care in Germany described above are reflected in the subjective perceptions of those affected. The results of the study may provide data-based starting points on improving the structural aspects of care for pwALS and their caregivers in Germany–in order to align care more closely with their needs and prevent harm. Furthermore, insights into potential associations between the perception of weak points of care and mental health, QoL, attitudes towards life-prolonging measures and physician assisted suicide and caregiver burden may provide political arguments to improve care structures in Germany toward a more holistic perspective, emphasising the above-mentioned palliative treatment goals.

### Study description and study design

**Fig. 2:**
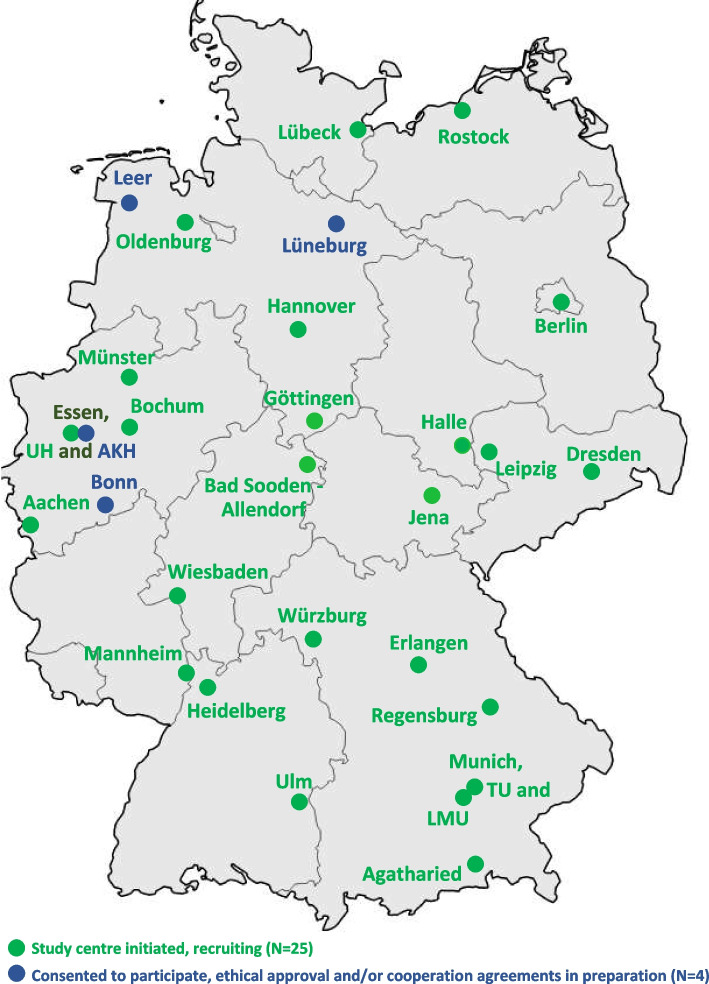
German ALS-centres involved in the project, mainly recruited from the German Network for Motor Neuron Diseases (MND-NET; https://mnd-net.de)

The aim of the study is to be achieved through a prospective, cross-sectional, multi-centre-study, based on paper-pencil-questionnaires for pwALS and their family caregivers. Study planning was conducted at the specialised outpatient clinic for motor neuron diseases of the University Hospital Carl Gustav Carus at the Technische Universität Dresden. Recruitment will mainly be done via the German Network for Motor Neuron Diseases (https://mnd-net.de), which our centre is a member of, potential collaboration partners were consecutively invited to contribute to the study. To date, 25 sites have already initiated and started recruitment, and another four sites have expressed informal commitments to participate in the study, with local ethical approval or cooperation agreement still pending (see Fig. 2). We aim to include a total of 500 pwALS by the end of 2024. Since this study has an explorative character, no statistical sample size calculation was conducted. The sample size is limited by the number of available patients who will be examined or treated in the participating ALS centres during the study period and who consent to study participation.

### Assessment instruments

The survey instruments for this study comprise four paper-pencil questionnaires, described in detail in Table 2.

**Table 2:** Description of the four questionnaires deployed in this study

	**Questionnaire for**	content	instrument
**(I)**	**ALS centres (to be filled out only once)**	patient volume; frequency of contacts; involvement of different professions	6 items
**(II)**	**Practitioners/treating neurologists**	disease onset	2 Items
		disease severity	ALS-FRS-R, Kings’ Staging
		use of NIV, IV or PEG	4 Items
		global functioning	Modified Ranking Scale
**(III)**	**Patients**	sociodemographic data	12 Items
		involvement of different professions in individual care	1 Item
		patients‘ needs in the six domains physical, psychological, social, spiritual, practical and informational and the corresponding professional support	Self-developed questionnaire, 44 items, 5-point-Likert-scale resp.
		improvement requests on professional care	13 Items
		knowledge about palliative care	2 Items
		attitudes towards potential life-sustaining measures (PEG, NIV and IV) and physician-assisted suicide	14 Items
		psychological well-being	HADS
		subjective QoL	McGill quality of life questionnaire
**(IV)**	**Family caregivers**	sociodemographic data	15 Items
		extend of involvement in patients‘ care and support	2 Items
		health issues or restrictions of professional activity, related to caregiving	2 Items
		need for professional support	CSNAT
		psychological well-being	HADS
		subjective QoL	McGill quality of life questionnaire
		caregiver burden	BSFC-s

*ALSFRS-R* Amyotrophic Lateral Sclerosis Functional Rating Scale, revised and consented German version, *BSFC-s* Burden Scale for Family Caregivers short version, *CSNAT* Carer Support Needs Assessment Tool, *HADS* Hamilton Anxiety and Depression Scale, *IV* Invasive ventilation, *NIV* Non-invasive ventilation, *PEG* Percutaneous endoscopic gastrostomy, *QoL* Quality of life

The section of the patient questionnaire that asks about patients’ needs and corresponding support was designed specifically for this project. Item selection was based on extensive literature search and pre-tested for comprehensibility.

Questionnaires for treating physician, patient and caregiver, related to a specific patient, are linked by a shared code that allows them to be assigned to each other.

### Recruitment of participants and eligibility criteria

As a first step, the treating neurologist personally informs eligible patients and their accompanying caregivers about the possibility to participate in the study during their outpatient or inpatient stay. If they provide consent, they receive detailed information and are subsequently enrolled in the study. Once consent has been obtained, participants receive the questionnaires to complete on their own and then send them back to the study centre Dresden, using postage-paid return envelopes. Patients who are physically unable to complete the questionnaire on their own are asked to seek assistance from a related person.

### Inclusion criteria

1. At least “possible ALS“ according to the revised El-Escorial-criteria (all disease stages; all subtypes)
2. Age of ≥ 18 years
3. No impairments of behaviour or mental performance relevant to everyday life that limit the ability to make judgments or give consent (e.g. as part of a comorbid frontotemporal dementia or another severe disease such as schizophrenia or delirium)

### Data analysis

The needs of patients in the above-mentioned six domains (Fig. 1) and their perception of professional care will be analysed descriptively. Additionally, the following research questions will be investigated:

- Are subjective deficiencies in professional support associated with psychological well-being and QoL, attitudes towards life-sustaining measures or physician-assisted suicide among pwALS or caregivers, or with caregiver burden?
- How frequently are non-medical actors (social workers, etc.) and SPC involved?
- Is subjective satisfaction with care associated with certain patient-side factors (e.g. gender, disease severity, urban or rural living environment)?
- Is subjective satisfaction with care associated with factors on the part of the ALS centres?

These questions will be investigated using correlation analysis and comparison tests (depending on level of data and statistical prerequisites). For statistical analysis we will use SPSS 23.0 software.

### Contacts

This study was initiated at the University Hospital Carl Gustav Carus at Technische Universität Dresden. PD Dr. med. habil. René Günther and Dr. rer. medic. Katharina Linse are PIs of the study. The project is funded by the German Society of Muscle Diseases (Deutsche Gesellschaft für Muskelkranke e.V.) and by a seed grant of the ALS association. Additionally, the study received support by ITF Pharma GmbH and Zambon GmbH.

### Perspectives

There is a large disparity between the multiple, complex and dynamic needs of pwALS and the German care structures for ALS, considering the palliative situation and the consequent focus on patients’ QoL. From a theoretical point of view and based on the experience of professionals engaged in ALS care, it appears evident, that there is a need for further development in ALS care. To the best of our knowledge, this study is the first systematic assessment of patients’ perception of and satisfaction with different domains of ALS care and of potential associations with mental health, QoL, attitudes towards life-prolonging measures and physician assisted suicide, and caregiver burden. Strengths of the study include its multi-centre approach, the potential of a high, nationwide caseload, and the possibility of deriving quantitative statements. It may provide valuable data on the perspectives of those affected, identifying the focal points that are most important to them and do have the largest impact on their wellbeing. The overriding goal is to design a concept that incorporates structural changes contributing to a more need-driven, whole-person, multidimensional and multiprofessional care for these vulnerable patients and their caregivers. This is essential to effectively match both the needs of pwALS and their caregivers, thereby avoiding insufficient of care as well as both mis- and overprovision.

## Data Availability

The data will be deposited on a protected server of the University Hospital Carl Gustav Carus at Technische Universität Dresden, access is strongly regulated. Reference lists and individual participant data will not be shared publicly. Upon reasonable request including a methodologically sound proposal for the usage of data approved by the responsible review committee, data may be shared.

## Abbreviations

ALS: Amyotrophic Lateral Sclerosis
ALSFRS-R: Amyotrophic Lateral Sclerosis Functional Rating Scale, revised and consented German version
BSFC-s: Burden Scale for Family Caregivers short version
CSNAT: Carer Support Needs Assessment Tool
HADS: Hamilton Anxiety and Depression Scale
IV: Invasive ventilation
NIV: Non-invasive ventilation
NPC: Neuropalliative care
PEG: Percutaneous endoscopic gastrostomy
pwALS: Patients with ALS
QoL: Quality of life
SPC: Specialised palliative care
